# Satisfaction with medical support in women with endometriosis

**DOI:** 10.1371/journal.pone.0208023

**Published:** 2018-11-29

**Authors:** Ilona Lukas, Alexandra Kohl-Schwartz, Kirsten Geraedts, Martina Rauchfuss, Monika M. Wölfler, Felix Häberlin, Stephanie von Orelli, Markus Eberhard, Bruno Imthurn, Patrick Imesch, Brigitte Leeners

**Affiliations:** 1 Department of Reproductive Endocrinology, University Hospital Zurich, Zurich, Switzerland; 2 Division of Gynecological Endocrinology and Reproductive Medicines, University Women’s Hospital, Berne, Switzerland; 3 Department of Psychosomatics, Charité Berlin, Berlin, Germany; 4 Department of Gynecology and Obstetrics, University Hospital Graz, Graz, Austria; 5 Department of Gynecology and Obstetrics, Canton Hospital St. Gallen, St. Gallen, Switzerland; 6 Department of Gynecology and Obstetrics, Triemli Hospital Zurich, Zurich, Switzerland; 7 Department of Gynecology and Obstetrics, Canton Hospital Schaffhausen, Schaffhausen, Switzerland; 8 Department of Gynecology, University Hospital Zurich, Zurich, Switzerland; Ordu University, TURKEY

## Abstract

Endometriosis affects various aspects of women’s lives. We searched for predictors for patient satisfaction with medical support (PSwMS) in women with endometriosis. The study was designed as a multi-centre retrospective cohort study. We approached women with histologically confirmed endometriosis from 2010 until 2016, comparing women satisfied to women dissatisfied with medical support. We analysed data on characteristics of endometriosis, PSwMS and the influence of disease characteristics on PSwMS. Information on satisfaction with medical support was collected through a standardized questionnaire. After exclusion of 73 women because of inchoately filled in questionnaires, data from 498 women was evaluated. Altogether, it was observed that 54.6% (n = 272) of the study participants were satisfied with medical support and 45.4% (n = 226) were not. Feeling adequately informed by the time of diagnosis (p < 0.001), taking women’s mental troubles seriously (p < 0.001) and supporting women in handling their pain (p < 0.001) were significantly associated with satisfaction.

We found adequate information to be the most distinctive indicator for PSwMS. Further, acknowledging psychological distress and supporting women in handling their symptoms rather than to alleviate them, positively affect PSwMS. To achieve PSwMS, healthcare providers have to give adequate information on endometriosis and its management.

## Introduction

Endometriosis is an oestrogen-dependent, chronic inflammatory condition, affecting between 5 and 10% of women in their reproductive years [1,2]. The symptoms of endometriosis include dysmenorrhea, dyspareunia, non-menstrual pelvic pain, dyschesia, dys-uria, musculoskeletal pain, fatigue and infertility [3–7]. Therefore, endometriosis affects women’s quality of life [1,8,9] and often leads to psychological strain [9].

However, women’s symptoms are not always taken seriously and are often normalized by doctors [10,11] or even by themselves [10]. Hence, many women suffer over years until they receive a reliable diagnosis [11]. In the UK, till today the diagnostic delay is 8.0 years (SD: 7.9) [12], in Austria and Germany 10.4 years (SD: 7.9) [13] and in the USA 11.7 years (SD: 9.1) [12].

Various therapeutic options for endometriosis as well as pregnancy show comparable and unfortunately very limited results [14,15]: Surgery has a positive effect on dyspare-unia, on pain and infertility as well as on quality of life on a short-term basis [16–18]. However, in 10-55% of the cases symptoms reoccur [19,20]. As medication e.g. mostly hormonal treatment often also does not succeed to reduce disease symptoms to a satisfactory degree, many women have to deal with chronic symptoms and consequently need medical support for a prolonged time [18]. The chronic nature of the disease, the diverse presentation as well as pain, infertility and fatigue, with its impact on private and professional areas of quality of life, challenge medical support [21,22]. But at the same time this makes adequate support particularly important.

Patient satisfaction with medical support (PSwMS) is essential since it does not only strongly influence quality of life and the psychological strain associated with endometriosis but is also related to an improving health status [23]. In addition, patient satisfaction reduces complaints and the number of second opinions sought of, potentially cutting costs [24]. However, very few studies have addressed satisfaction with medical support in endometriosis [11]. These studies had very small sample sizes (30 participants) and did not systematically search for options for improvement.

With the present study, we therefore aimed to (i) get an overview on patients’ satisfaction with medical support in women with endometriosis, with a careful differentiation between women attending self-help groups and those recruited in hospitals and private offices. Also, we (ii) searched for predictors for PSwMS. We (iii) investigated the provided information through medical professionals and perceived attitude of doctors toward patients to adapt current support structures better to patients needs. Than, we considered the individual needs of women with endometriosis. Lastly, we (iv) analysed their suggestions for adequate medical support.

## Methods

### Study design

The study was designed as a multi-centre retrospective cohort study on quality of life in women diagnosed with endometriosis [6,25,26]. The local ethics committee (Cantonal Ethics committee Zurich, Switzerland, KEK_StV-Nr. 05/2008) approved the study. All women included in this analysis, signed an informed consent, including the permission to collect data from medical charts for confirmation of diagnosis. The study was conducted according to the declaration of Helsinki.

The STROBE criteria were used to draft the manuscript [27].

### Study participants

The questionnaire was given to women diagnosed with endometriosis. They were recruited in Switzerland, mainly at the university hospital Zurich, the Triemli hospital Zurich, and the hospitals in Schaffhausen, St. Gallen, Winterthur, Baden, Solothurn and Walenstadt, as well as in associated private offices. The Charité Berlin, the Albertinen hospital Hamburg, the Vivantes Humbold Klinikum Berlin and the University hospital Aachen also made an essential contribution in Germany. In Austria, women were recruited at the university hospital in Graz. In addition, women were approached at different self-help groups in Germany.

### In- and exclusion criteria

Women were only included if the diagnosis of endometriosis was histologically and/or surgically confirmed. Participants also needed the mental, psychological and linguistic ability to understand and answer the questions. They were excluded if the stage of endometriosis was unknown, the questions about PSwMS (Were you satisfied with medical support in regard to endometriosis?) remained unanswered or if more than 50% of the answers investigating details of patient satisfaction were missing.

### Recruitment

Women with endometriosis were approached by medical staff, their gynaecologist or within the self-help group and received information about the study. With their consent to participate, women were handed the study documents, containing an informational flyer about the progression, confidentiality, and aims of the study, as well as a questionnaire and a return envelope. Furthermore, a declaration of consent was included.

### Questionnaire

Specialists for endometriosis and psychosomatic medicine from the universities of Zurich and Berlin developed the questionnaire, in cooperation with the leading board of the endometriosis self-help groups in Germany. For this study, we included questions on sociodemographic data (Table 1) and questions on satisfaction with medical support (Table 2).

**Table 1:** Sociodemographic characteristics and potential confounders.

** **	**Women satisfied with** **medical support** **(n = 272) n (%)**	**Women dissatisfied with medical support** **(n = 226) n (%)**	**p-value**
**Age (mean in years ± SD)**			
**Total study group (n = 497)**	37.3 ± 7.1	38.5 ± 7	0.071^a^
WrHPO (n = 432)	37.1 ± 7.2	37.3 ± 6.8	0.757^a^
WfSHG (n = 65)	40.6 ± 5.3	42.9 ± 5.7	0.146 ^a^
**Nationality (n = 495)**			
**Total study group**			<0.001^b^
Swiss	132 (48.5)	69 (30.5)	
German	110 (40.4)	131 (58)	
Austrian	5 (1.8)	3 (1.3)	
Others	25 (9.2)	20 (8.8)	
**WrHPO (n = 430)**			0.059^b^
Swiss	132 (48.5)	69 (30.5)	
German	92 (33.8)	85 (37.6)	
Austrian	5 (1.8)	3 (1.3)	
Others	25 (9.2)	19 (8.4)	
**WfSHG (n = 65)**			0.533^b^
Swiss	0 (0)	0 (0)	
German	18 (6.6)	46 (20.4)	
Austria	0 (0)	0 (0)	
Others	0 (0)	1 (0.4)	
**Marital status (n = 495)**	** **	** **	
**Total study group**			0.17^b^
Married/ long-term relationship	232 (85.3)	180 (79.6)	
Single	40 (14.7)	43 (19)	
**WrHPO (n = 430)**			0.181^b^
Married/ long-term relationship	216 (79.4)	141 (62.4)	
Single	38 (14)	35 (15.5)	
**WfSHG (n = 65)**			0.555^b^
Married/ long-term relationship	16 (5.9)	39 (17.3)	
Single	2 (0.7)	8 (3.5)	
**Time since initial diagnosis** (n = 465)	** **	** **	
**Total study group**			0.022^c^
0-12 months	83 (30.5)	49 (21.7)	
13-60 months	106 (39)	89 (39.4)	
61-120 months	43 (15.8)	44 (19.5)	
>121 months	18 (6.6)	33 (14.6)	
**Total study group median, in months**			
(n = 465)	24.5	39	<0.001^c^
**WrHPO median, in months**			
(n = 400)	22.5	34.5	0.026^c^
**WfSHG median, in months**			
(n = 65)	50.5	83	0.093^c^
**Stage (n = 498)**	** **	** **	
**Total study group**			0.772^c^
Stage I	41 (15.1)	41 (18.1)	
Stage II	62 (22.8)	41 (18.1)	
Stage III	74 (27.2)	69 (30.5)	
Stage IV	95 (34.9)	75 (33.2)	
**WrHPO (n = 433)**			0.443^c^
Stage I	41 (15.1)	38 (16.8)	
Stage II	59 (21.7)	31 (13.7)	
Stage III	68 (25)	57 (25.2)	
Stage IV	86 (11.6)	53 (23.5)	
**WfSHG (n = 65)**			0.538^c^
Stage I	0 (0)	3 (1.3)	
Stage II	3 (1.1)	10 (4.4)	
Stage III	6 (2.2)	12 (5.3)	
Stage IV	9 (3.3)	22 (9.7)	
**Number of interventions (median)**			
Total study group (n = 467)	1	2	<0.001^c^
WrHPO (n = 402)	1	2	0.008 ^c^
WfSHG (n = 65)	2	2	0.171^c^
**Difficulties conceiving (N = 310)**			
Total study group	115 (42.3)	104 (46)	0.403^b^
WrHPO	105 (38.6)	79 (35)	0.819 ^b^
WfSHG	10 (3.6)	25 (11.1)	0.228^b^
**Chronic pain caused by** **endometriosis (N = 433)**			
Total study group	115 (42.3)	138 (61.1)	<0.001^b^
WrHPO	101 (37.1)	100 (44.2)	0.001^b^
WfSHG	14 (5.1)	38 (16.8)	0.231 ^b^

a: p-value based on an independent t-test analysis

b: p-value based on a Pearson Chi-Square test analysis

c: p-value based on a Mann Whitney test analysis

**Table 2:** Satisfaction with medical support in relation to addressing key endometriosis-associated symptoms (n = 498).

	**satisfied (% (n))**^a^	**Dissatisfied (% (n))**^a^	**was not necessary** **(% (n))** ^a^	**p-value**^b^
**The desire of having children was addressed at:**				
**Total study group**				
the time of diagnosis	36.9	20.1	16.7	<0.001
a later date	28.1	25.7	26.5	0.005
**WrHPO**				
the time of diagnosis	35.5	16.5	14.5	<0.001
a later date	26	19.8	23.3	0.003
**WfSHG**				
the time of diagnosis	1.4	3.6	2.2	0.999
a later date	2.2	5.8	3.2	0.884
**Pain therapy was addressed at:**				
**Total study group**				
the time of diagnosis	23.3	5.6	8.4	<0.001
a later date	20.3	13.3	15.3	<0.001
**WrHPO**				
the time of diagnosis	22.3	4.8	7.8	<0.001
a later date	18.5	10.8	14.7	<0.001
**WfSHG**				
the time of diagnosis	1	0.8	0.6	0.122
a later date	1.8	2.4	0.6	0.094
**Mental health support was addressed at**				
**Total study group**				
the time of diagnosis	6.2	1.2	16.7	<0.001
a later date	8.0	6.2	17.9	<0.001
**WrHPO**				
the time of diagnosis	6	1	16.5	<0.001
a later date	6.4	4.2	17.6	<0.001
**WfSHG**				
the time of diagnosis	0.2	0.2	0.2	0.643
a later date	1.6	2	0.2	0.116

a: Percentages were calculated in relation to the total number of participants (n = 498)

b: p-value based on a Pearson Chi-Square test analysis

As there are no validated tools to evaluate medical support in the context of endometriosis questions on satisfaction with such support were designed for this study. First, women had to report whether they were satisfied with medical support provided by health care providers (yes/no). For closer investigation, we analysed the influence of chronic pain and infertility at the initial consultation, when diagnosis was explained, as well as at a later time point. We also addressed mental health support, since women with endometriosis are more likely to experience anxiety and depressive symptoms, which need treatment to prevent a manifestation of affective disorders [28].

We then evaluated provided information and education about endometriosis during medical consultation (Table 3). For this assessment, we asked 20 questions and classified them into four groups: Communication, interpersonal, expertise and therapy. With a Likert scale reaching from 1 (is not true at all) to 7 (is entirely true), we assumed a median of 3 or less means the modality was neglected. Questions on satisfaction with medical support were pilot tested in a group of 30 women with endometriosis for reasons of understanding and accuracy. Women participating in the pilot study were not included in the final analysis.

**Table 3:** Assessment of provided information and education. Comparing medians.

**The doctors**	**Satisfied** **Median**	**Dissatisfied** **Median**	**p-value**^a^
***Communication***			
**took sufficient time to explain**			
Total study group	6	3	<0.001
WrHPO	7	3	<0.001
WfSHG	4	2	0.01
used a well comprehensible language to explain the condition			
Total study group	6	3	<0.001
WrHPO	6	4	<0.001
WfSHG	5	3	0.001
provided enough time to answer my questions			
Total study group	7	3	<0.001
WrHPO	7	4	<0.001
WfSHG	5	3	<0.001
***Interpersonal***			
**saw me as a whole human being with body and mind**			
Total study group	6	2	<0.001
WrHPO	6	2	<0.001
WfSHG	4	2	0.008
**took my mental troubles seriously**			
Total study group	6	2	<0.001
WrHPO	6	2	<0.001
WfSHG	4	2	<0.001
**considered me as an equal interlocutor**			
Total study group	6	2	<0.001
WrHPO	6	2	<0.001
WfSHG	4	2	0.001
**took me and my complaints seriously**			
Total study group	7	3	<0.001
WrHPO	7	4	<0.001
WfSHG	6	3	<0.001
***Expertise***			
**provided competent support**			
Total study group	6	3	<0.001
WrHPO	6	3	<0.001
WfSHG	6	3	<0.001
**advised me well with regard to fertility**			
Total study group	6	3	<0.001
WrHPO	6	3	<0.001
WfSHG	4	3	0.048
**supported me handling my pain**			
Total study group	6	2	<0.001
WrHPO	6	3	<0.001
WfSHG	4.5	2	<0.001
**cooperated well with other doctors e.g. pain-specialists, to reduce my pain**			
Total study group	3	1	<0.001
WrHPO	3	1	<0.001
WfSHG	2	1	0.008
**provided adequate sexual counselling**			
Total study group	4	1	<0.001
WrHPO	4	1	<0.001
WfSHG	2	1	0.033
**were open to consider a second or third opinion**			
Total study group	5	3	<0.001
WrHPO	5	3	<0.001
WfSHG	4.5	2.5	0.001
***Therapy***			
**presented a realistic perspective on curing endometriosis**			
Total study group	6	3	<0.001
WrHPO	6	3	<0.001
WfSHG	5.5	3	<0.001
**informed me fully about conventional therapeutic options**			
Total study group	6	3	<0.001
WrHPO	6	2	<0.001
WfSHG	5.5	3	0.002
**informed me fully about additional therapeutic options**			
Total study group	4	2	<0.001
WrHPO	4	2	<0.001
WfSHG	3.5	1	0.001
**were open towards alternative methods**			
Total study group	4	2	<0.001
WrHPO	4	2	<0.001
WfSHG	3.5	1	0.011
**considered my own experience in dealing with endometriosis in medical decisions**			
Total study group	6	2	<0.001
WrHPO	6	2	<0.001
WfSHG	5	2	<0.001
**advised me to exchange with other affected women**			
Total study group	2	1	<0.001
WrHPO	2	1	<0.001
WfSHG	2	1	0.031
**developed a plan for the best possible life with endometriosis**			
Total study group	3	1	<0.001
WrHPO	3	1	<0.001
WfSHG	2	1	<0.001

The scale was from 1 to 7, women rated 7 if they agreed and 1 if they disagreed.

a: P-value is based on a Mann Whitney test analysis

With a preselected list of six answers and the possibility to add a free text answer, we evaluated sources women used to get information about endometriosis and which source they perceived most helpful.

Finally, women were given the opportunity to suggest improvements for supporting and educating women with endometriosis in free text answers. Their suggestions were analysed by content one by one and resulted in 14 different subsections, which are shown in Table 4. All information could be integrated into these 14 points.

Answers from women participating in self-help groups (WfSHG), were compared to responses from women recruited in hospitals or private offices (WrHPO) to evaluate whether particular dissatisfaction with medical support might have been the motivation to participate in a self-help group.

**Table 4:** Suggestions to improve medical support (n = 265).

	**Patients**
1) Better education about endometriosis through doctors, gynaecologists or brochures, including information about cause and healing	52
2) Create a treatment plan adjusted to suit personal preferences and requirements including alternative treatment, treatment for symptoms and psychological issues	42
3) Taking women and their pain seriously	35
4) Doctors should be better qualified or take further training	28
5) Doctors should suggest information centres, endometriosis associations, lectures or self-help groups	28
6) Doctors should take more time to explain endometriosis and talk to the patient about it	28
7) More information about treatment possibilities and process, e.g. pain treatment, operations and treatment after operations	21
8) There should be more information about endometriosis in public and schools	17
9) Timely diagnosis, early detection and evaluation	17
10) More sensitivity from doctors regarding infertility	8
11) Better communication among doctors	7
12) Positive thinking without raising false hopes	7
13) Pay more attention to symptoms and individual issues	5
14) More information about sexuality	4

### Statistics

All statistical analysis was performed with SPSS, Version 22 for Mac OS IBM. An independent t-test for continuous variables and a Mann Whitney-U-test for ordinal-scaled variables evaluated differences between satisfied and dissatisfied women. We compared mean values to evaluate provided information and education to adjust statistical outliers. Categorical characteristics were compared through Pearson Chi-Square. A p-value < 0.05 was considered statistically significant. We conducted a stepwise multiple logistic regression analysis to identify factors predicting PSwMS. We included sociodemographic characteristics with significant differences between satisfied and dissatisfied women, the information on feeling adequately informed about endometriosis at the time of diagnosis as well as the answers to any topic addressed during consultations (see Table 2). In addition, answers to the questions from Table 3: “Took my mental troubles seriously”, “Took me and my complaints seriously”, “Advised me well in regard to fertility” and “Supported me handling my pain” were entered into the analysis, because they were considered as clinically very important.

## Results

A total of 573 questionnaires were returned. We had to exclude 73 because of incomplete data. We collected 67 of the 573 questionnaires in self-help groups, of which 65 could be included in the analysis. Lack of time and the intimate nature of some of the questions were the most common reasons to decline study participation.

Of all participants, 54.6% (n = 272) were satisfied with their medical support and 45.4% (n = 226) were dissatisfied. Only 27.7% (n = 18) of WfSHG were satisfied. Table 1 shows an overview about the sociodemographic data of the study participants.

Women reporting to be adequately informed by the time of diagnosis were significantly more often satisfied with medical support than women feeling inadequately informed (p < 0.001). A total of 85.2% (n = 231) satisfied women and 21.4% (n = 48) of the dissatisfied women felt adequately informed at the time of initial diagnosis. In WfSHG we also found significant differences for provision of adequate information at the time of diagnosis (satisfied = 38.9%; dissatisfied = 12.8%; p = 0.018). However, in satisfied WfSHG the inadequate informed women were predominant. In WrHPO, we found adequate information in 84.2% (n = 224) of satisfied and in 23.7% (n = 42) of dissatisfied women. There were also significant differences (p-value <0.001).

Table 2 shows if topics with impact on quality of life (e.g. pain management, fertility) and mental health support were part of medical counselling as well as the importance of women diagnosed with endometriosis attributed to each issue.

With one exception, more than 80% of women rated all three topics (pain management, fertility, mental health support) as a necessary part of medical counselling. Women were significantly more often satisfied with medical support when each of these three issues was addressed either at initial diagnosis or later. In WfSHG we found no significant difference in mentioning these topics between satisfied and dissatisfied women.

Provided information and education about endometriosis as well as its association with patients’ satisfaction is summarised in Table 3.

Each question showed a significant difference between satisfied and dissatisfied women in the total study group, in WrHPO and in WfSHG. The weakest result was on the advice to exchange with other affected women, followed by doctors cooperating well with other doctors, e.g. pain-specialists and doctors developing a plan for the best possible life with endometriosis.

Overall, most medians were lower in WfSHG when compared to the total study group and most medians from WrHPO were as high as medians from the total study group or higher. The three questions with the lowest result in the total study group also had a very low result in WrHPO and in WfSHG. In addition, in WfSHG the question about sexual counselling had a median of 2 in satisfied women and a median of 1 in dissatisfied women. An exception in WrHPO made the question about information on conventional therapeutic options, which had a median of 2 in dissatisfied women compared to a median of 3 in the whole study group and WfSHG.

The content analysis of womens’ suggestions for improvement of medical support revealed 14 different aspects, which are presented in Table 4.

The need for better education and information, including information about the cause and healing was mentioned by 10.4%. Furthermore, 8.4% requested an individual approach to deal with endometriosis including alternative treatment and treatment for psychological issues and 7% wanted doctors to take women and their pain seriously.

**Fig 1:**
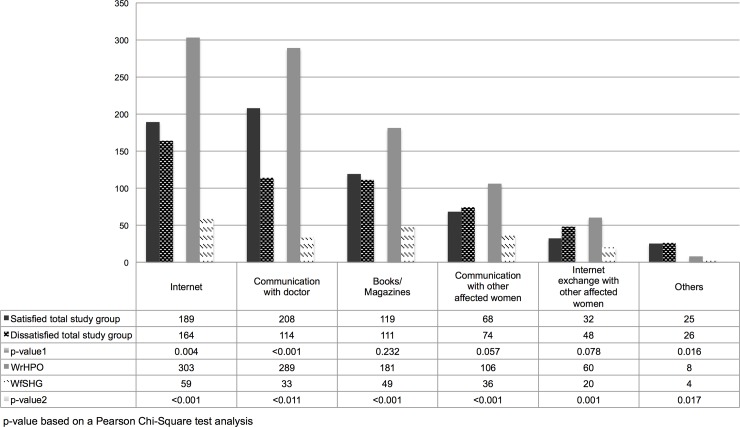
Sources for information gathering. Multiple answers were possible. P-value1: satisfied vs. dissatisfied total study group; p-value2: satisfied vs. dissatisfied WrHPO; p-value3: satisfied vs. dissatisfied WfSHG. Internet: n = 362; Communication with doctor: n = 322; Books/Magazines: n = 230; Communication with other affected women: n = 142; Internet exchange with other affected women: n = 80; Others: n = 62.

Fig 1 shows the sources satisfied and dissatisfied women used to gather information on endometriosis.

**Fig 2:**
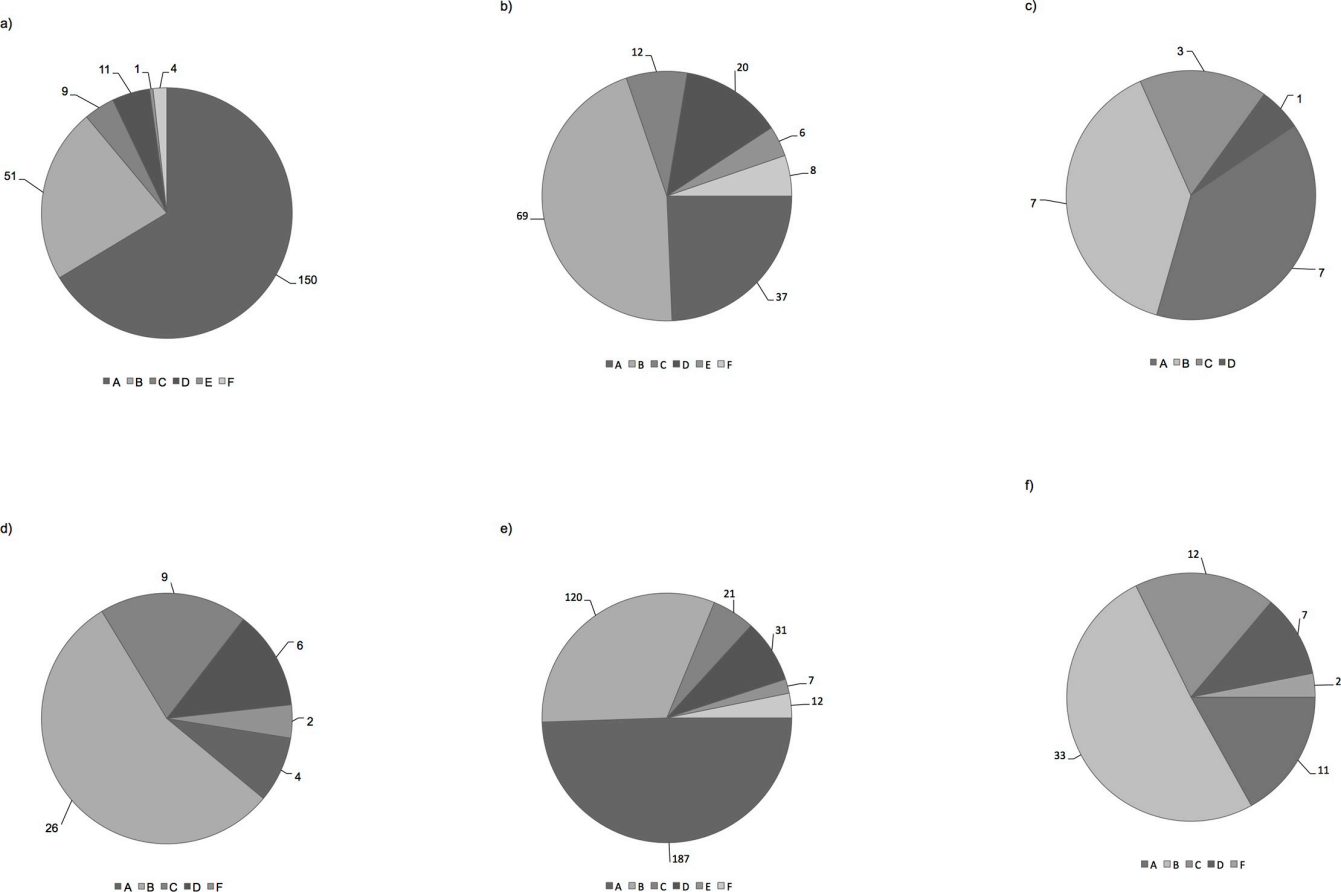
**a-f: Sources experienced most helpful to gather information.** a) in satisfied WrHPO (n); b) in dissatisfied WrHPO (n); c) in satisfied WfSHG (n); d) in dissatisfied WfSHG (n); e) in WrHPO; f) in WfSHG (n); A: Communication with doctors (n = 198); B: Internet (n = 153); C: Books/Magazines (n = 33); D: Exchange with affected women (n = 38); E: Online exchange with affected women (n = 7); F: Others (n = 14).

Fig 2 gives an overview on the sources women experienced as most helpful to gather information on endometriosis. Sources of helpful information varied significantly between satisfied and dissatisfied women of the total study group (p < 0.001; p-value based on a Pearson Chi-Square test analysis), in WrHPO (p < 0.001) and between WrHPO and WfSHG (p < 0.001) but not in WfSHG only (p = 0.056). Satisfied women estimated communications with doctors more often as beneficial, while dissatisfied women preferred the Internet. In both groups, these two sources were used most often.

In Table 5 the regression analysis shows the predictors we found for patient satisfaction. Even though all factors taken into the regression analysis showed statistically significant differences, only three factors could be identified as predictors for PSwMS. Provision of adequate information increases the probability for satisfaction almost eight times. Furthermore, taking mental troubles seriously and supporting women in handling their pain improved satisfaction with medical support.

**Table 5:** Regression analysis.

Predictors for PSwMS	p-value	Exp(B)	95% C.I. for Exp(B)	
			Lower	Upper
Felt adequately informed by the time of diagnosis	**<0,001**	**7.862**	**3.960**	**15.607**
Took my mental troubles seriously	**<0.001**	**1.429**	**1.175**	**1.736**
Supported me in handling my pain	**<0,001**	**1.784**	**1.439**	**2.211**

Hosmer and Lemeshow Test Chi-square=2.804; df=7, p-value=0.902

## Discussion

In this study, satisfied women did not significantly differ from dissatisfied women in age. Two previous studies found higher patient satisfaction in older patients, especially those over 65 years [29,30], so the average age under 45 in our study could explain differences in findings. More than half of Swiss and Austrian women were satisfied with medical support, while only 45.6% of German participants were satisfied, which likely has to be attributed to the WfSHG within the German group. As a greater proportion of WfSHG are affected by pain and problems in conceiving, we assume that WfSHG present a more severely affected group of women and tend therefore to be less satisfied. Operative results positively influence PSwMS [31]. This is in agreement to our findings in the total study group and WrHPO, in which we found a lower average of interventions in satisfied women. However, WfSHG do not differ significantly in number of surgeries when comparing satisfied to dissatisfied women, both groups had an average of 2 interventions. As to expect, women with a shorter duration of the disease were more likely to be satisfied. Especially in WfSHG our findings show fewer satisfied women and more time since initial diagnosis passing. This is in line with previous findings [30], showing an increase of satisfaction over time only on women with few or no disease symptoms.

In line with previous studies [31–34], women with chronic pain were significantly more often dissatisfied with medical support. The comparison between WrHPO and WfSHG emphasises this outcome. However, regression analysis did not confirm the absence of chronic pain as a predictor for PSwMS, eventually because endometriosis-associated pain is expected and met patients’ expectations are a predictor for PSwMS [30,35]. Another factor might be successful treatment of pain [23]. Patients distinguish between quality of care and quality of treatment [36], which could be another reason why the support in handling pain is a predictor of PSwMS, but in our study chronic pain itself is not. In a disease like endometriosis, this gives health professionals the possibility to achieve PSwMS through supporting and listening to patients’ needs, even though pain can not be alliviated. Earlier findings showed the necessity for better education about infertility [37] and since women with endometriosis have a lower monthly fecundity [4], which is in agreement with women’s appreciation of doctors mentioning fertility at the time of diagnosis or at a later date. Further, pain is known as one of the major concerns in women with endometriosis [22]. Therefore, it is not surprising that women who were asked about pain management were more often satisfied. The association between endometriosis, depression and anxiety [28] supports that mental health support is associated with PSwMS in our study and those of others [38]. Psychological support can help to handle pain, which in reverse can be an additional cause for depressions [22]. Therefore, pain therapy and mental health support should always be a priority in patient care.

We found significant results when investigating PSwMS in relation to addressing key endometriosis-associated symptoms in the total study group and WrHPO but not in WfSHG, which is probably a result of the limited sample size of WfSHG. The assessment of provided information and education shows vast differences in satisfied and dissatisfied women, with particularly insufficient information reported by WfSHG. Using a comprehensible language avoids language barriers and misunderstandings [37], which underlines the higher satisfaction when doctors offered enough time to give and clarify information. The experience not to be taken seriously [10,11,23], was found to be a relevant issue in dissatisfied women and also in WfSHG. For our participants, the solution is more awareness in public and in health professionals, which has been recommended by the scientific community for at least ten years [37]. Most doctors were reported not to be open to second or third opinions, an experience confirmed by others [11]. Also, our participants criticized lack of cooperation with other specialists. As endometriosis affects different aspects of life and various medical fields, an interdisciplinary approach would help to better adjust treatment to patients’ needs. Although dyspareunia is an essential symptom of endometriosis, there are several strategies to live a fulfilling sexuality despite endometriosis, therefore, sexuality should be addressed [38,39]. Sexuality was a neglected topic even in satisfied women of our study and studies from other authors [40]. Our results indicate, that infertility is adressed more often, but not sufficiently.

Information on therapeutic options was reported to be unsatisfactory, which will at least partly be due to the difficult prediction of the individual development of the disease as well as individual effects and side effects of the treatment [3,21]. In addition, doctors did not follow a holistic approach to reduce consequences of endometriosis on quality of life. Furthermore, doctors were described as not supporting women in exchanging experiences with other affected women, even though it has been reported as helpful [37]. Another aspect for improvement is the lack of information on additional and alternative therapeutic options. As it is crucial to inform women about all possible therapies to let them participate in medical decisions and allow them gain control on disease management [37], a structured information guide on characteristics of the disease and contacts of institutions, specialists and self-help groups could be beneficial.

The need for more information on treatment became even more evident when looking at the free text answers, where women expressed the need for more information in all sections of endometriosis education. In agreement with a Dutch study [41], they had the impression, that only part of the doctors are adequately trained in endometriosis. According to their reasoning general practitioners only see a limited number of women with endometriosis and therefore have difficulties to interpret disease symptoms correctly. According to the free text answers women want more information, they want to be taken seriously and awareness towards endometriosis should be raised. Direct communication was reported a valuable resource to increase PSwMS in a previous study [42]. In accordance, satisfied women from our study valued communication with doctors as most helpful, while dissatisfied women used the Internet to increase their knowledge and improve their strategy to deal with endometriosis. This becomes particularly evident when comparing WrHPO to WfSHG. While almost half of WrHPO found communication with doctors most helpful, this way of obtaining information if only at third place in WfSHG. In contrast, half of WfSHG estimated the internet as most helpful.

Our regression analysis confirmed adequate information by the time of diagnosis to be a predictor for PSwMS besides supporting women in handling their pain, therefore the first consultation may determine whether a woman will be satisfied with medical support or not. According to a previous study, being well informed about a chronic disease supports coping [37], indeed it is crucial to achieve PSwMS [31]. As endometriosis is connected with emotional distress, feelings of isolation, guilt, worry, worthlessness and hopelessness [43], the relevance of taking mental troubles seriously was an expected outcome. Even though treating physicians may lack specific training [41], trying to understand and showing empathy can already help to cope [42].

The strengths of our study are the big sample size and the inclusion of only women with a surgically/histologically confirmed diagnosis. Also, we carefully differentiated between women participating in self-help groups and women recruited in hospitals or private offices. Furthermore, in some questions women had the possibility to express the imperative they attributed to certain topics. Whereas, the assessment of provided information and education only evaluates whether or not a modality was mentioned during a doctors’ appointment. It does not show how women rated the importance of each item. PSwMS changes over time [30]. Hence, women who were dissatisfied in this study could be satisfied when asking at a later date and vice versa. Also, they might not recall all information they were given and therefore recall bias could occur. Recall bias could also result from the level of satisfaction with medical support and the study design allows no reliable statement on causal effects.

## Conclusion

With this study we could show that many women with endometriosis are still not satisfied with medical support. Most women were lacking information about the disease or treatment options. We identified adequate information as an indicator for patient satisfaction. Further, many women experienced psychological distress and acknowledged that it positively affects PSwMS. Last, we found that it is more important to support women in handling their symptoms than to alleviate them to enhance satisfaction.

To achieve PSwMS, we have to take the time to inform and educate patients during the first consultations but also in consecutive ones. It is crucial to show all treatment options to let women adequately participate in therapeutic decisions. Meeting patients with empathy to create a relationship, where they feel welcome to ask questions or present their specific needs and individual expectations is mandatory for PSwMS. Such approach is essential to allow endometriosis-affected women to achieve the best possible quality of life. Delicate topics like sexuality should be integrated, symptoms and psychological strains acknowledged, and interdisciplinary support offered when needed and wanted.
